# Editorial: Women in skin cancer vol II: 2022

**DOI:** 10.3389/fonc.2023.1253081

**Published:** 2023-08-08

**Authors:** Selma Ugurel, Sapna P. Patel

**Affiliations:** ^1^ Department of Dermatology, University Hospital Essen, University of Duisburg-Essen, Essen, Germany; ^2^ German Cancer Consortium (DKTK), Partner Site Essen/Düsseldorf, Essen, Germany; ^3^ MD Anderson Cancer Center, The University of Texas, Houston, TX, United States

**Keywords:** diagnostics, prognostics, personalized treatment approaches, immunotherapy, targeted therapy

## Introduction

Skin cancer remains a significant public health concern worldwide, with its incidence on the rise in recent years. However, advancements in molecular biology have led to a better understanding of its spectrum of different entities. Moreover, research and technological developments have opened up new horizons in the treatment of these malignancies. This Research Topic explores clinical topics surrounding skin cancer, including diagnostics, prognostics, and new personalized treatment approaches in skin cancer management around the globe.

## Diagnostics

Artificial intelligence algorithms have demonstrated remarkable potential in analyzing skin images and assisting in the diagnosis of skin cancer. Machine learning models can analyze large datasets and accurately differentiate between benign and malignant lesions, aiding clinicians in making informed decisions. The article of Kriegsmann et al. describes how the authors localized and categorized skin tumors on whole formalin-fixed paraffin-embedded tissue slides without prior annotation. For this aim, they previously trained a convolutional neuronal network on major non-tumor anatomical tissue structures of the skin as well as the most relevant skin tumor categories. Subsequently, they validated their system on an external test set of tissue slides with very good results: Automated differentiation of BCC, SCC, melanoma, naevi and non-tumor tissue structures was possible, and a high diagnostic accuracy was achieved in the validation (98%) and test (97%) set. Most importantly, the research team openly provided all images and codes they used for their project to enable other researchers to improve and validate their data.

## Prognostics

The field of melanoma prognostics has developed notable advancements in recent years, with the integration of advanced technologies and sophisticated predictive models. Molecular profiling techniques, including gene expression profiling and next-generation sequencing, offer a deeper understanding of the molecular landscape of melanoma, allowing for more precise prognostication, though their use is not fully validated for integration into clinical algorithms at this time. Nevertheless, these approaches enable the identification of gene signatures associated with aggressive disease and may provide insights into potential therapeutic targets. Moreover, machine learning algorithms, by analyzing large datasets and incorporating multiple prognostic factors, facilitate the development of robust prognostic models. The integration of these advanced tools holds immense promise for enhancing prognostication in melanoma and aiding in clinical decision-making. Augustin et al. investigated a large set of 4,790 diabetic patients with cutaneous melanoma for their melanoma recurrence rates, progression free survival (PFS), and overall survival (OS) with and without exposure to metformin, an anti-diabetic drug that has been shown to reduce intratumoral hypoxia, improve T-cell function, and increase the sensitivity to PD-1 blockade in pre-clinical studies. The authors found a reduction in recurrence rate, overall survival, and interestingly also incidence of brain metastasis in patients who received metformin, with the caveat that this is merely an association and not necessarily a correlation finding. These results suggest rationale for ongoing and future clinical trials studying the potential augmentation of checkpoint blockade with metformin in advanced melanoma.

## Personalized treatment approaches

Treatment options for skin cancer have evolved beyond traditional methods, with a focus on personalized approaches that consider individual characteristics and genetic factors.

## Immunotherapy

One of the most remarkable breakthroughs in skin cancer treatment is the advent of immunotherapy. This revolutionary approach harnesses the power of the immune system to target and destroy cancer cells. Immune checkpoint inhibitors targeting PD1, CTLA4 and other checkpoint molecules, have demonstrated exceptional efficacy in treating advanced melanoma as well as other skin cancer entities. These medications block the proteins that suppress the immune response, allowing immune cells to recognize and attack cancer cells effectively. Immunotherapy has not only shown remarkable response rates but also provided durable remissions and improved overall survival in patients. However, these potent agents also lead to severe side effects, which often limit their further use in affected patients. In this regard, Neuville et al. report a case of capillary leak syndrome associated with a chylothorax in a melanoma patient who has been treated with adjuvant nivolumab (anti-PD1). The knowledge on early recognition and adequate management of these adverse events therefore is of high importance for the clinical care of advanced skin cancer patients. Helbig and Klein give an overview on the treatment of pleomorphic dermal sarcomas with immune checkpoint inhibitors. Their review demonstrates that these rare skin tumors respond well to immunotherapy due to their high tumor mutational burden, which is linked to their suggested UV-induction, as well as their high number of tumor-infiltrating lymphocytes. Lodde et al. report on COVID-19 vaccination which led to unimpaired seroconversion in advanced skin cancer patients under treatment with either immunotherapy or targeted therapy. Notably, an impaired serological response was observed in patients who were immunocompromised due to concomitant diseases or previous chemotherapies, whereas immunosuppressive comedication due to severe adverse events did not impair the serological response to COVID-19 vaccination.

## Targeted therapy

Targeted therapies have transformed the management of specific types of skin cancer. In cases where melanomas harbor specific genetic mutations such as BRAF or NRAS, targeted drugs like vemurafenib, cobimetinib, dabrafenib and trametinib can be used. These medications specifically inhibit the abnormal signaling pathways that drive cancer growth, leading to tumor regression and improved patient outcomes. The advent of precision medicine and molecular profiling has revolutionized the selection of appropriate targeted therapies, allowing for personalized treatment strategies tailored to each patient’s genetic profile. Shaikh et al. report on a phase-1 clinical trial combining targeted therapy with vemurafenib plus cobimetinib with the PD1 inhibitor pembrolizumab in patients with BRAF-mutant metastatic melanoma. Nine of 30 planned patient were treated and responded well with an objective response rate of 78% (7/9). However, significant adverse events of CTCAE grade 3 to 4 were observed in 8 of 9 patients, leading to an early closure of this study. Salman et al. demonstrate that despite the clear benefits of targeted therapies for BRAF-mutated melanoma in other regions of the world, there is no clear path to prepare Latin Americans for a sustainable personalized medicine approach. Melanoma therefore represents an increasing public health burden with extensive unmet needs in Latin America, a problem which must be solved in the future. Zattarin et al. report a patient with advanced extramammary Paget’s disease showing a long lasting benefit from the HER-2 inhibitor trastuzumab, indicating that this approach is worth exploring further in the management of this rare skin cancer entity.

## Conclusion

The field of skin cancer research and management is rapidly evolving, with new and hot topics continuously emerging. New methods and techniques for diagnostics and prognostics, as well as personalized treatment approaches are at the forefront of research and development. Through these advancements, we hope to further improve patient outcomes, reduce the burden of this disease, and enhance collaborative efforts in combating skin cancer.

## Author contributions

Both authors contributed equally to this work drafting, editing, and approving the final version of the manuscript.

